# Use of Modern Contraception by the Poor Is Falling Behind

**DOI:** 10.1371/journal.pmed.0040031

**Published:** 2007-02-06

**Authors:** Emmanuela Gakidou, Effy Vayena

**Affiliations:** 1 Harvard Initiative for Global Health, Harvard University, Cambridge, Massachusetts, United States of America; 2 Institute for Quantitative Social Science, Harvard University, Cambridge, Massachusetts, United States of America; 3 Department of Reproductive Health and Research, World Health Organization, Geneva, Switzerland; Johns Hopkins Bloomberg School of Public Health, United States of America

## Abstract

**Background:**

The widespread increase in the use of contraception, due to multiple factors including improved access to modern contraception, is one of the most dramatic social transformations of the past fifty years. This study explores whether the global progress in the use of modern contraceptives has also benefited the poorest.

**Methods and Findings:**

Demographic and Health Surveys from 55 developing countries were analyzed using wealth indices that allow the identification of the absolute poor within each country. This article explores the macro level determinants of the differences in the use of modern contraceptives between the poor and the national averages of several countries. Despite increases in national averages, use of modern contraception by the absolute poor remains low. South and Southeast Asia have relatively high rates of modern contraception in the absolute poor, on average 17% higher than in Latin America. Over time the gaps in use persist and are increasing. Latin America exhibits significantly larger gaps in use between the poor and the averages, while gaps in sub-Saharan Africa are on average smaller by 15.8% and in Southeast Asia by 11.6%.

**Conclusions:**

The secular trend of increasing rates of modern contraceptive use has not resulted in a decrease of the gap in use for those living in absolute poverty. Countries with large economic inequalities also exhibit large inequalities in modern contraceptive use. In addition to macro level factors that influence contraceptive use, such as economic development and provision of reproductive health services, there are strong regional variations, with sub-Saharan Africa exhibiting the lowest national rates of use, South and Southeast Asia the highest use among the poor, and Latin America the largest inequalities in use.

## Abstract 2

Analysis of demographic and health surveys from 55 developing countries confirms the increasing use of modern contraceptives, but their use by people living in absolute poverty lags considerably behind.

## Editors' Summary

**Background.:**

Access to safe and effective methods of contraception is seen by many to be a basic human right. Contraception plays an important role in improving women's health (by reducing the risks that would otherwise accompany unwanted births), as well as the social and financial situation of women and their families. However, despite a steady increase in contraceptive use worldwide over the past few decades, the World Health Organization says there is still a significant unmet need for birth control. Very many women worldwide, probably around 123 million, would like to limit the number of children that they might have but, despite this, they are not using contraception. There are probably many factors responsible for this unmet need, including the availability of health services, a woman's level of education, her social and financial situation, and cultural factors.

**Why Was This Study Done?:**

Although it is clear that use of contraception has been increasing worldwide over the past few decades, particularly in developing countries, it is not clear whether the poorest people in each country have also benefited from this trend. Given that contraception has important effects on health and on the financial and social circumstances of a family, it is important to find out whether there are any differences in contraceptive use between the poorest and richer members of society.

**What Did the Researchers Do and Find?:**

This research project was based on data collected by a survey oganization about various aspects of the health, social, and economic status of households worldwide. Over 100 surveys conducted between 1985 and 2003 were used from the publicly available survey database. The researchers then classified each household for which there was survey information as being in the poorest 20% of households or not, worldwide. Importantly, this categorization reflects whether the household was in the “absolute poor” worldwide, not just the poorest for their respective country. Since information about household income was not directly available from the surveys, the researchers had to use an approach based on ownership of consumer goods and services (referred to as “asset-based wealth measures”). The researchers then looked at trends in contraceptive use amongst the poorest households, and examined whether contraceptive use was linked to other factors, such as level of education and average income.

The data showed that use of contraception by poor women was linked to the overall degree of poverty in the woman's country. Poor women from countries where many households were in the poorest 20% worldwide were far less likely to use contraception. Secondly, the researchers found that poor women were less likely to use contraception than average women in their country, and in richer countries, there seemed to be a larger gap in contraceptive use between “average” and “poor” women. Finally, the researchers found that various factors were linked to greater contraceptive use, which included the date of the survey (more recent surveys were more likely to show greater use of contraceptives), the wealth of the country where the survey was done (richer countries showed greater use), and whether women had skilled birth attendants (a marker of access to reproductive health services, and again this pointed to greater use of contraceptives). However, the researchers did find that there was huge variability in use of contraceptives worldwide, even when comparing countries at a similar economic level.

**What Do These Findings Mean?:**

This study shows that although contraception use is increasing over time, its use by poor people is low. The gap in use of contraception between poor people and “average” people also seems to be increasing over time and is wider in richer countries. The reasons behind these findings are not clear, but the data suggest that nations and international health organizations need to focus their attention on providing contraceptive services in a way that will reach people who have very low incomes.

**Additional Information.:**

Please access these Web sites via the online version of this summary at http://dx.doi.org/10.1371/journal.pmed.0040031.

- The World Health Organization's pages on family planning provide evidence-based guidance, official publications, and information on family planning research
- There is a helpful Wikipedia entry on birth control (note that Wikipedia is an internet encyclopedia that anyone can edit)
- The United Nations Population Fund collects data and helps countries to understand and analyze population trends, and its Web site provides an overview of population issues. It also publishes a yearly report, "State of World Population"
- There are also many independent not-for-profit organizations that focus on reproductive health and population issues. One example is Interact Worldwide, and the "links" page on this organization's Web site lists the sites of many other organizations active in this area
- The US Centers for Disease Control and Prevention includes relevant data and statistics via its global reproductive health minisite

## Introduction

The use of safe and effective methods of contraception allows couples to determine the number and spacing of their pregnancies. Access to such methods was deemed a fundamental human right by the 1994 International Conference on Population and Development (ICPD)—a forum in which countries committed to work toward achieving the goal of universal access to reproductive health services, including access to effective contraceptives. Improving the use of effective contraception contributes to reducing the burden of reproductive ill health by decreasing mortality and morbidity of unwanted pregnancies [1,2]. Further, increasing contraceptive use reduces fertility, which, in turn, can play a crucial role in poverty reduction [3,4].

The widespread increase in the use of contraception is one of the most dramatic social transformations of the second half of the twentieth century [5]. Spurred by the international population control movement in the 1960s, 1970s, and 1980s, contraceptive use increased dramatically throughout the developing world [6]. This increase is likely due to multiple factors including access to modern contraception. Such access, in turn, is likely related to micro- and macroeconomic factors, including women's education, household income, integration into the modern economy, and to the proactive efforts of governments and other health providers to make contraceptive services available. A question that has not been addressed to date is whether the poor have also experienced this positive trend, which has been demonstrated for national average use rates [7,8].

The first Millennium Development Goal is the reduction in absolute poverty. The development community has increasingly focused its attention on the circumstances of the absolute poor living on $1 a day and the near-poor living on $2 a day [9]. Given the international commitment to ensuring that couples are able to exercise their right to plan their pregnancies and the important role of contraceptive use in promoting both reproductive health and economic growth, it is essential to determine whether the absolute poor in different parts of the world are able to use modern contraception. In short, is the apparent global progress in the use of modern contraceptives also benefiting the poorest?

To answer this question and provide evidence for monitoring progress toward international goals, the analysis presented here uses data from the Demographic and Health Surveys (DHS) [10], as well as a methodology identifying the absolute poor women within each country, to explore differences in progress between the rich and the poor and the macro level determinants of these differences.

## Methods

### Data

The findings of this analysis are based on an analysis of 110 DHS [10]. The DHS is a household survey program that collects data on maternal and child health, using nationally representative samples of women of reproductive age. Table 1 presents the countries, years, and sample sizes of the surveys used in this analysis. The surveys span a period of nearly 20 years (1985–2003) and include several countries with three or more surveys from Latin America and the Caribbean, sub-Saharan Africa, and South and Southeast Asia.

**Table 1:**
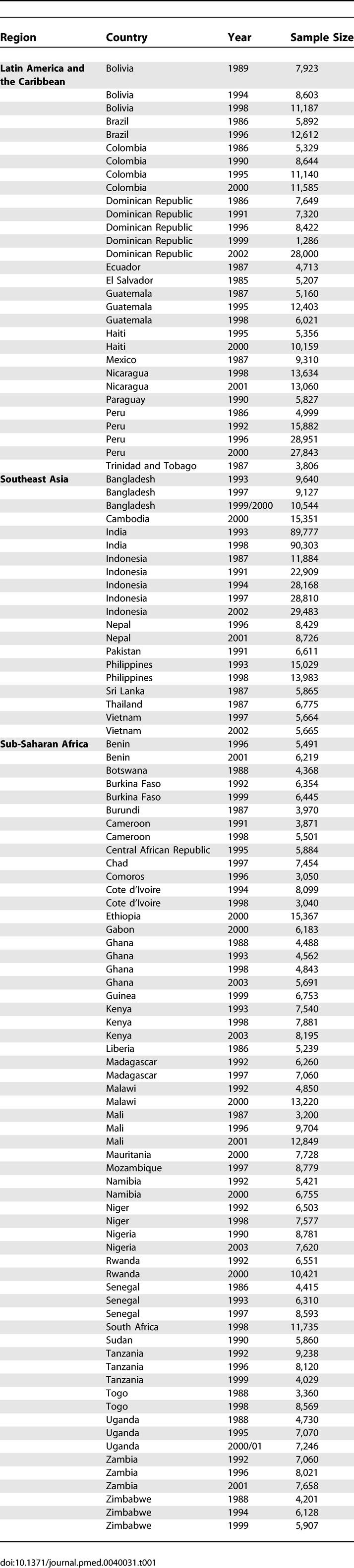
List of Surveys Included in the Analysis

### Dependent Variable

The use of contraception is analyzed for nonpregnant, ever-married women in the age group 15–49 y. We limit the analysis to ever-married women (married, divorced, or widowed), because in several countries the DHS samples only within that group. Only modern contraceptive methods are included due to their higher efficacy compared to traditional methods [11]. These methods are: injectable and oral hormones, implants, intrauterine devices, spermicides, condoms, diaphragms, female sterilization, and male sterilization. The dependent variable in the analysis is the percent of ever-married women using modern contraceptive methods, measured for each quintile of wealth in each survey.

### Explanatory Variables

To examine factors that might potentially influence contraceptive use across countries and over time, regressions were run using the STATA statistical package, version 9.2 (http://www.stata.com/). Estimates of socioeconomic characteristics, skilled birth attendance, and education are directly derived from the DHS datasets, using the sampling weights. Average income per capita in international dollars is available from the Penn World Tables and the World Bank [12,13] for multiple years for each country. We used the estimate of GDP per capita for the year of the survey that was used in the analysis. The Gini index was available for 1990 and 2000; the year that was closest to the survey year was included in the study [13]. The Gini index measures the extent to which the distribution of income across households within a country is unequal; it takes on values from 0 to 100 with 0 representing perfect equality and 100 perfect inequality.

### Estimation of “Wealth Quintiles”

We measured wealth using a method developed by Ferguson et al. [14]. As wealth is a latent variable that cannot be directly observed, we estimate it using information on predictors of wealth (age, education, sex of the household head, and urban/rural location) and indicators of wealth (electricity, radio, television, refrigerator; bicycle, motorcycle, car; main construction materials of the walls, roof, and floor of the house; source of drinking water and type of toilet facility, as well as, in some cases, other country-specific assets). The set of assets available in the DHS is limited, as the questionnaire was not designed to be used for the estimation of a wealth index.

A detailed discussion of the statistical methods applied to estimate the index can be found elsewhere, and its application seen in other studies [14–18]. Briefly, a random-effects probit model was used to identify “cutpoints” that represent the point on the wealth (latent) scale above which a household is more likely to own a particular asset. This “asset ladder” was then applied to every household in each survey to produce adjusted estimates of household wealth. Linear regression of asset cutpoints from all surveys was used to place the wealth estimates from all surveys on the same scale, thus leading to a wealth index that is directly comparable across surveys. The correlation between the average economic status at the country level and gross domestic product (GDP) per capita is 0.83 across all DHS surveys.

For this analysis, we have distributed the population in each country to “developing country quintiles.” These quintiles have been constructed so that quintile 1 refers to the bottom 20% of the population across all developing countries, taking into account DHS sampling weights and the relative population size of each country. Thus, quintile 1 does not represent the bottom quintile in each country but can be thought of as a measure of absolute deprivation. This construction of quintiles across all surveys allows for comparisons and analyses of variations in contraceptive use rates in the most deprived.

The composition and the percentage of the population in absolute deprivation changes over time within a country, as some countries have achieved a reduction in the proportion of the population living in absolute poverty over the past two decades. Despite improvements in the levels of poverty, the gap between the national average and those in the poorest quintile in key health indicators remains of critical policy significance. A central dimension of the effectiveness of a government is its ability to deliver services to those in absolute deprivation. As the national income grows, a country's capacity to do this should increase. The construction of quintiles in a comparable way over time and across countries allowed us to monitor the use of modern contraceptive methods by the poor over time.

### Regression Analysis

We explored two types of regression models: (i) ordinary least squares (OLS) regression, which was run separately for each wealth quintile and the national average; (ii) seemingly unrelated regression, which was applied to the five quintiles at the same time, to control for potential correlation of the error terms. In all regressions, the dependent variable was the percent use of modern contraceptive methods, and the independent variables were GDP per capita, Gini coefficient, year of survey, percent births attended by skilled personnel, average years of education, and a regional dummy variable.

Our findings were robust to the choice of model. In the tables and figures that follow, we chose to present the estimates from the OLS regressions, as the seemingly unrelated regressions can be applied only to countries with estimates of contraceptive use for all quintiles of income, which reduces the sample size. As the choice of model does not affect the substantive conclusions on the size and significance of the effects, we present the results from the OLS model.

## Results

Average rates of use of modern contraceptive methods have increased over the past few decades in most developing countries. Our findings illustrate the differences in use by the poorest groups and how these differences relate to the estimated national average use.

Figure 1 presents a graphical exploration of the use of modern contraceptive methods by women in the poorest quintile, compared to the percentage of women in each country in that quintile. As the proportion of women in the bottom quintile increases, the estimate of use by the bottom quintile approximates the national average. Figure 1 shows, as expected, that the prevalence of modern contraceptive use by poor women is strongly related to the proportion of women in each country who are poor. The striking result from Figure 1 is that there are large variations in modern contraceptive use across countries with similar proportions of women in poverty; for example, in countries with 30% of women in the bottom quintile, the prevalence of use ranges from near zero to 24%.

**Figure 1:**
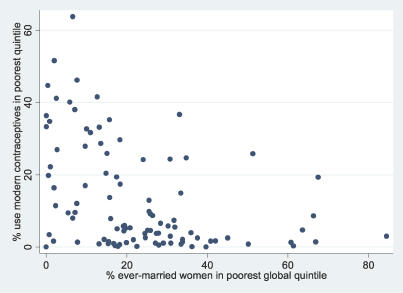
Modern Contraceptive-Method Use in the Poorest Global Quintile The relationship between the percentage of ever-married women and the use of modern contraceptive methods by ever-married women in the poorest global quintile is shown.

Figure 2 summarizes the relationship between the use of modern contraceptives and the level of economic development. As income per capita increases, the gap between the poorest members of the population and those representing the national average appears to increase. The positive slope in Figure 2 implies that as countries become richer, the poor remain impoverished. Among the data in Figure 2 are Mexico and Gabon (not indicated specifically), which have a comparatively high level of economic development with relatively small gaps in contraceptive prevalence between the national average and the poor. Figure 2 also highlights that at a given level of economic development, there are large variations across countries in the gaps in modern contraceptive use by the poor.

**Figure 2:**
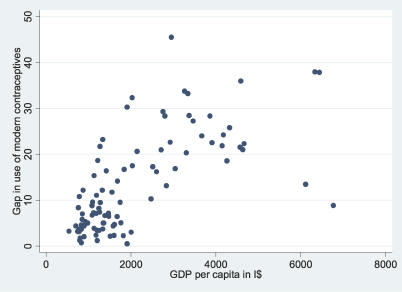
Modern Contraceptive Use and Level of Economic Development Gaps increase at higher incomes. The gap in modern contraceptive use rates between the national average and the poorest quintile versus GDP per capita in international dollars is shown.

To establish whether or not the poorest are being left behind in the use of modern contraceptive methods across the set of countries in our analysis, a systematic examination of the relationships is presented in Table 2, which shows the results of three multivariate regressions. The regressions use data from each survey and attempt to formalize the relationships between the use of modern contraceptives and potential macro level determinants. The three regressions explore relationships between the explanatory variables and (i) national average level of use of modern methods, (ii) use by the poorest quintile, and (iii) the gap in use between the average and the poorest.

**Table 2:**
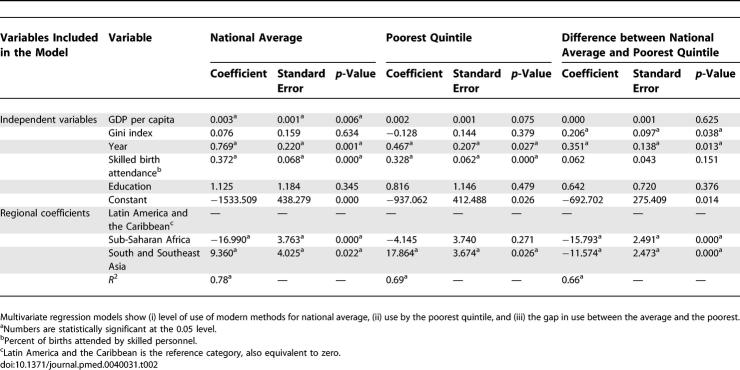
Results from Multivariate Regression Models

At the national level, average income per capita, year of the survey, and percent of births attended by skilled personnel are all significantly associated with higher levels of modern contraceptive use. The variable representing the year of the survey is included to capture the secular trend in use of modern contraceptives, which has been shown to be increasing over time. Skilled birth attendance can be considered as a crude proxy for access to reproductive health services, and its statistical significance implies that, controlling for all other factors in the model, it is strongly associated with higher levels of contraceptive use [19,20]. Income inequality and education of women do not seem to be significant predictors of average levels of contraceptive use. Table 2 shows that even after controlling for these macro level determinants, strong regional differences remain with South and Southeast Asian countries having the highest average levels of use, followed by Latin America and the Caribbean, and sub-Saharan Africa at the lowest average use levels.

The results for quintile 1 are markedly different from those at the national level. Skilled birth attendance and year are the only significant variables in the model, while GDP per capita and education are not. This implies that if skilled birth attendance is acting as a proxy for supply of (or access to) services, it is highly significant not only for the national average but also for the poorest populations. The coefficients on the regional effects in this regression are striking. South and Southeast Asia are the regions that show significantly higher rates of modern contraception in the absolute poor, on average 17% higher use rates than the poor in Latin America. Rates of use by the poorest quintile in Latin America and sub-Saharan Africa are not statistically distinguishable, despite significantly higher average use rates in Latin America.

The last column in Table 2 shows the results for the regression of the gap between the national average and the poorest. The year of the survey is statistically significant, suggesting that, controlling for the level of economic development, over time the bottom quintile is doing worse relative to the mean. Higher levels of income inequality are also associated with larger gaps between the national average and the poorest. The finding that countries with higher economic inequality also exhibit higher inequalities in modern contraceptive use is not surprising, but it is important, implying that inequalities in contraceptive coverage reflect overall inequalities in a country. As in the other two regressions, the regional coefficients suggest significant differences across the regions. Latin American countries exhibit significantly larger gaps in use between the poor and the average, while compared to Latin America sub-Saharan African countries have on average a gap that is smaller by 15.8% and Southeast Asian countries have on average 11.6% smaller gaps. This result implies that, even after controlling for variables that might be important in determining the use of contraception, there is a strong regional effect, with Latin America showing the largest inequalities in contraceptive use.

The *R* ^2^ coefficients shown in Table 2 suggest that the multivariate regressions explain a large amount of observed variation across countries in the national averages (78%) and high, but smaller, amounts of the differences across the poorest quintile (69%), and the gaps (66%).

Finally, Figure 3 shows the multivariate regression coefficients and 95% confidence intervals for all quintiles for skilled birth attendance and year of survey. The data in Figure 3 imply that the effect of skilled birth attendance rates is significant and at roughly the same magnitude across the bottom four quintiles. Put differently, the supply of reproductive health services provided by the health system of each country is an important determinant of rates of modern contraceptive use and is similarly important for women across levels of wealth. The only group in which the effect of skilled birth attendance is not statistically significant is women in the top wealth quintile. This suggests that national investments in reproductive health services benefit the majority of the population and are positively associated with higher rates of modern contraceptive use.

**Figure 3:**
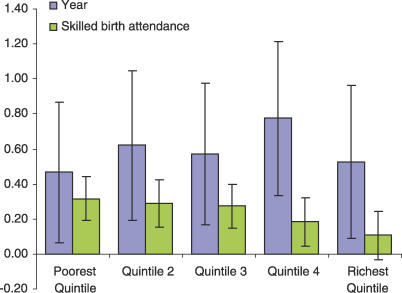
Multivariate Regression Coefficients and 95% Confidence Intervals for All Income Quintiles for Skilled Birth Attendance and Year of Survey

The coefficient on the year of the survey is significant for all quintiles. This variable is used as a proxy for the secular trend in contraceptive use that remains after controlling for the effect of economic development, income inequality, level of education, and urbanization. This finding suggests that the increases in contraceptive use seen over time are benefiting women in all quintiles of wealth. Furthermore, in combination with the findings presented in Table 2 that the gap between the poor and the national average is increasing over time, it suggests that the poorest women are exhibiting a slower rate of increase in the use of modern contraceptives than the rest of the population, and if this trend continues, they will continue to be left behind with regard to contraceptive use.

## Discussion

Consistent with expected relationships for demand and access, the few studies undertaken to date with a focus on socioeconomic issues indicate that contraception rates are lower in poor countries and, within the limited set of countries analyzed, lower in poor women [21–25]. This study demonstrates that despite increases in national averages over time, the use of modern contraception by the absolute poor remains low, and the gaps in use across wealth quintiles persist and are increasing.

The result of this study—that the gap in modern contraceptive prevalence between the absolute poor and the rest of the population in developing countries is increasing over time and tends to widen in countries with higher incomes—needs careful exploration. Is this difference driven largely by the relationship between demand for modern contraception and economic status, or by trends and relationships related to the availability of contraceptive services, or both? At both the micro and macro levels, there is a strong relationship between modern contraception rates and economic status. This is likely due to complex pathways relating income to both the demand for and also the supply of contraceptive services. The gap in modern contraceptive use could be getting larger, because as national income per capita rises, the gap in income between the rich and the absolute poor is also rising. Given the relationship between income inequality and contraception, increasing gaps in income might be driving the increasing gaps in contraception. Another explanation of the increasing gap may be that as countries get richer, the proportion of the population living in absolute poverty is decreasing. It is possible that the composition of the poorest quintile is becoming increasingly “selective” to include the most disadvantaged and hardest-to-reach populations.

These observations, however, should not lead to complacency about accepting as inevitable that the absolute poor will always lag behind in contraceptive use. While several studies have evaluated the impact of geographical, educational, social, cultural, and political factors on the use of contraception [26–34], the findings of this analysis can be interpreted as showing that modern contraception rates in the absolute poor vary greatly across countries and are highly sensitive to the availability of services. The persistent gap between the absolute poor and the rest of the population is unlikely to be due to low demand for fertility regulation in these households. Countries could in principle differentially increase contraception rates in the absolute poor through increased provision of services that are tailored to local circumstances and financially accessible. We argue that the steadily increasing gap, in combination with greater national income inequalities, is a question of political priority for contraception and more broadly for reproductive health services.

The secular trend toward reduced levels of total fertility at any given level of national income has been well documented [35]. This reduction in fertility has been attributed in part to cultural change, as well as to changing economic and social status of women, increasing access to information, and the role of mass media. This analysis showed that for all wealth quintiles there is a statistically significant increase in contraception rates over time. This trend may be a reflection of the nexus of cultural and social transformation. While improvements are seen across all quintiles, the gap between countries' average use and use by the poor has been increasing over time. The mechanisms of cultural change, such as exposure to mass media and changing socioeconomic roles for women, may simply not be having much influence on those in absolute deprivation [30]. Regardless of the reason for the differential time trend, it implies that with each succeeding decade, inequalities in modern contraceptive use will increase unless some active policies to counteract this trend are pursued.

An important policy issue is the responsiveness of modern contraceptive prevalence to the availability or supply of contraceptive services. Careful analysis of this at the local level requires disentangling demand from a range of provider attributes including price, quality, cultural sensitivity, physical distance, and language. Direct measures of supply or availability are difficult to construct for cross-country comparisons. Skilled birth attendance rate may be considered as a crude proxy for the availability of selected reproductive heath services [36]. Attended deliveries at a given level of income will be higher where the financial, physical, cultural, and other barriers are lower. If the interpretation of skilled birth attendance as a measure of supply is valid, the results demonstrate that contraceptive prevalence is highly sensitive to the supply of services. In fact, the bottom four quintiles appear to be equally sensitive to the availability of services. This finding is consistent with analyses that have emphasized considerable unmet need for contraceptive services [31] and studies that illustrated that contraceptive use increases as more types of methods become available [37,38]. If modern contraceptive prevalence in the absolute poor can be significantly increased by enhanced supply, it highlights that the widening gap between rich and poor could be avoided through targeted interventions [39]. Improvements of the supply of services will be more effective if they take into consideration the reasons for differential uptake of modern contraception by the poor where they are available.

This study reaffirms the substantially lower levels of modern contraceptive prevalence in all income groups in sub-Saharan Africa, and to a lesser extent Latin America and the Caribbean, as compared to South and Southeast Asia [40]. Although the roles of religion, traditional concepts of family formation, health concerns, and medical barriers in demand for modern contraception have been explored [29,30,33], this analysis provides no insight into these patterns. It does, however, point out that these regional factors interact with economic status in such a way that the gaps between rich and poor are much larger in Latin America than in other regions. This gap remains even after taking into consideration supply (as approximated by skilled birth attendance), income per capita, income inequality, and secular trends. This finding highlights that Latin American health systems may need to pay particular attention to policies that affect delivery of reproductive health services to the absolute poor.

### Limitations

Interpretation of these findings must take into consideration several limitations of this study. The analysis was undertaken for ever-married women only. Although in some countries modern contraceptive use by unmarried women could be substantial, this information is not available for a large number of DHS whose sampling frame includes ever-married women only, and where these data are available for unmarried women, there are concerns about the degree to which underreporting of contraceptive use due to cultural and social concerns may undermine the validity of the data. While the DHS program focuses a considerable amount of resources in making sure that the data are of high quality, the present analysis has relied on self-reported use of contraception, which suffers from several limitations, similar to other indicators measured through self-reports [41].

The wealth index has been constructed using all available indicator variables in the DHS. The DHS were not designed to measure economic status, and therefore the set of items included in this part of the questionnaire is not ideal for differentiating households throughout the distribution of income in a country. Further investments in more accurate measurement of income and identification of the absolute poor are necessary in the coming decades of reporting toward the Millennium Development Goals. A final limitation worth mentioning is that the relationships explored in this analysis are at the national level, and much could be learned by a more in-depth study of individual level determinants. Even across countries, however, a considerable amount of the variation in the use of modern contraception by the poor and the gaps with the national average use rates remains unexplained by the macro level determinants included in this analysis. Further study into factors that might explain the remaining variation could provide insight into interventions and policies that would be effective at reducing the gaps. For example, information on the level of financial commitment to family planning as a percentage of public and total expenditure on health is not available for most countries; however, it might provide insight into how the level of financial investment influences uptake of modern contraception by the poor.

### Conclusions

As national income per capita rises, countries have increasing fiscal capacity to finance the delivery of services to the absolute poor. Taking advantage of this fiscal space requires, of course, the social and political commitment to use scarce resources to improve the circumstances and opportunities of the poor. This study shows that if current patterns persist, the absolute poor will continue to be left behind in the overall progress in increasing modern contraceptive use. On the positive side, it appears that making reproductive health services available is a powerful determinant, all other things being equal, of contraceptive prevalence. A concerted effort by governments to facilitate an increase in physical, financial, and cultural access to reproductive health services for the poor could have a major effect. The fundamental challenge will be to raise the international and national priority accorded to reproductive health services for the poor. Paradoxically, in an era of increased resource flows for global health through mechanisms like the Global Fund for AIDS, Tuberculosis and Malaria and GAVI (the Global Alliance for Vaccines and Immunisations), contraceptive use and related reproductive health services seem increasingly difficult to place on the health agenda [42–44]. The trends that have been observed to date provide strong evidence that without new priority attention to modern contraception, the poor will remain deprived of the fundamental right to its demonstrated benefits.

## Abbreviations

DHS: Demographic and Health Survey(s)
GDP: gross domestic product
OLS: ordinary least squares
